# Influence of pathogen and focus of infection on procalcitonin values in sepsis patients with bacteremia or candidemia

**DOI:** 10.1186/s13054-018-2050-9

**Published:** 2018-05-13

**Authors:** Daniel O. Thomas-Rüddel, Bernhard Poidinger, Matthias Kott, Manfred Weiss, Konrad Reinhart, Frank Bloos, for the MEDUSA study group

**Affiliations:** 1 Center for Sepsis Control and Care, Jena University Hospital, Jena, Germany; 2 Department of Anesthesiology and Intensive Care Medicine, Jena University Hospital, Am Klinikum 1, 07747 Jena, Germany; 3 Department of Anaesthesiology and Intensive Care Medicine, University Hospital Schleswig-Holstein, Campus Kiel, Kiel, Germany; 4 Clinic of Anaesthesiology, University Hospital Medical School, Ulm, Germany

**Keywords:** Procalcitonin, Gram-negative bacteria, Gram-positive bacteria, Sepsis, Bacteremia, Focal infection

## Abstract

**Background:**

This study aimed to evaluate the accuracy of procalcitonin (PCT) serum concentrations to diagnose Gram-negative bacteremia and the association of PCT serum concentrations with more specific pathogens and the focus of infection.

**Methods:**

Secondary analysis of the prospectively collected patient-level dataset from a cluster randomized quality improvement trial was performed. The trial included sepsis patients with organ dysfunction treated in the participating intensive care units from 2011 to 2015. Test performance for the prediction of Gram-negative bacteremia was assessed by receiver operating curve analysis. Independent effects of specific pathogen groups and foci of infection on PCT concentrations were assessed by linear logistic regression models.

**Results:**

Blood cultures (BC) and PCT concentrations had been taken in 4858 of 6561 documented patients. PCT was significantly higher in Gram-negative bacteremia compared to Gram-positive bacteremia or candidemia (*p* < 0.001). The area under the curve was 0.72 (95% confidence interval 0.71–0.74) for the prediction of Gram-negative bacteremia compared to all other blood culture results including negative blood cultures. The optimized cutoff value was 10 ng/ml (sensitivity 69%, specificity 35%). PCT differed significantly between specific groups of pathogens (*p* < 0.001) with highest concentrations in *Escherichia coli*, *Streptococcus* species and other *Enterobacteriaceae*. PCT was highest in urogenital followed by abdominal infection and lowest in respiratory infection (*p* < 0.001). In a linear regression model, Streptococci, *E. coli* and other *Enterobacteriaceae* detected from BC were associated with three times higher PCT values. Urogenital or abdominal foci of infection were associated with twofold increased PCT values independent of the pathogen.

**Conclusions:**

Serum PCT concentrations are higher in patients with Gram-negative bacteremia than in patients with Gram-positive bacteremia or candidemia. However, the discriminatory power of this difference is too low to guide therapeutic decisions. Variations in PCT serum concentrations are not determined solely by Gram-negative or Gram-positive bacteria but are also affected by distinct groups of pathogens and different foci of infection.

**Trial registration:**

ClinicalTrials.gov, NCT01187134. Registered on 23 August 2010.

**Electronic supplementary material:**

The online version of this article (10.1186/s13054-018-2050-9) contains supplementary material, which is available to authorized users.

## Background

The production of procalcitonin (PCT), a prohormone of calcitonin, is upregulated in ubiquitous tissues in response to inflammatory stimuli including severe infection [1, 2]. Even though elevated PCT serum concentrations are not exclusively specific to infections, PCT is considered among the best available biomarkers to diagnose sepsis [3] and can be helpful in reducing antibiotic exposure [4]. Beside initiation or discontinuation of antimicrobial therapy, the potential role of PCT in choosing specific antimicrobial substances without microbiological proof has been discussed [5, 6]. Indeed, several studies have reported higher PCT levels in Gram-negative bacteremia compared to Gram-positive bacteremia [5, 7–10]. However, as interpretation of these studies is hampered by small sample size or limited clinical data, we analyzed data from a previous quality improvement trial in patients with severe sepsis or septic shock with a high number of patients [11]. The aim of this retrospective analysis was to evaluate the accuracy of PCT serum concentrations to predict Gram-negative bacteremia and to analyze whether specific pathogens and the focus of infection have a relevant influence on PCT serum concentrations.

## Methods

### Study design

This is a secondary analysis of the prospectively collected patient-level dataset from the MEDUSA study, a cluster randomized quality improvement trial aiming to improve early sepsis diagnosis and treatment in the participating hospitals by a multifaceted educational program [11]. The original trial was registered at ClinicalTrials.gov (NCT01187134) and was approved by the local ethics committees at each participating institution (see Additional file 1 for a complete list) and by the responsible state data protection boards.

### Study population

Patients treated between July 1, 2011 and May 31, 2015 on the participating intensive care units (ICUs) with proven or suspected infection and at least one new infection-related organ dysfunction were eligible for inclusion. Patients were excluded if they had relevant limitations of therapy, were not treated on a participating ICU or had infection control measures started at another hospital before transfer.

### Data collection and laboratory diagnostics

Data collection and definitions were as described previously [11, 12]. Briefly, onset of severe sepsis or septic shock was defined as the time of first infection-related organ dysfunction. PCT measurements were performed in local laboratories using commercially available assays as part of routine care. Highest values of PCT and other laboratory parameters and most pathological values for vital signs but not changes over time were recorded within the first 24 h after the onset of severe sepsis as baseline data.

Blood cultures were drawn at severe sepsis onset before or after the start of antimicrobial therapy and processed and analyzed according to local standards. Blood culture results reporting typical contaminants (e.g., coagulase-negative Staphylococci, *Corynebacterium* species, *Propionibacterium acnes* and other skin colonizers) were carefully assessed by the treating physicians and if judged as contaminations were considered blood culture-negative in all analyses. Only isolates considered real pathogens were reported. Pathogens were grouped into Gram-positive bacteria, Gram-negative bacteria and candida. Pathogens were further divided into seven groups according to their phylogenetic relationship (*Staphylococcus* spp., *Streptococcus* spp., *Enterococcus* spp., *Escherichia coli*, *Enterobacteriaceae* other than *E. coli*, *Pseudomonas* spp. and *Candida* spp.). An eighth group was composed of all rarely detected pathogens that did not belong to one of the seven groups. The focus of infection was identified retrospectively by the treating physician taking into account all available clinical and microbiological data. For analysis, foci of infection were grouped into four categories (respiratory, abdominal, urogenital and bones/soft tissue).

### Data analysis

Categorical data are expressed as absolute and relative frequencies, continuous data as median and interquartile range (IQR). Differences between groups were assessed by chi-square test or Kruskal–Wallis test. To assess the classification performance of PCT to predict Gram-negative bacteremia, receiver operating characteristic (ROC) curves with area under the curve (AUC) were calculated. In a second step, optimal cutoff values were determined by the Youden’s index and test performance measures were calculated. In order to achieve normal distribution for further analysis, PCT was logarithmically transformed to the base of 10 (logPCT) and distribution was assessed by P–P plots (see Additional file 2: Figure S1). To look for subgroups of pathogens and foci of infection distinguished by PCT values, one-way ANOVA with Scheffe and Dunnet’s T3 post hoc comparison were performed based on logPCT.

We calculated linear logistic regression models with logPCT as the dependent variable in order to assess for an independent effect of pathogen groups and foci of infection on PCT values. Specific groups of pathogens, foci of infection and interaction terms were stepwise included in the model. To account for potential confounding by antimicrobial therapy, the models were restricted to cases with blood cultures drawn before the start of a new antimicrobial therapy. In a further approach, potential confounders were added to the model by forward selection.

*p* ≤ 0.05 was considered statistically significant for all tests. Estimated values are presented with 95% confidence intervals (CIs). All analyses were performed using IBM SPSS Statistics 23.0 and 25 (IBM, Armonk, NY, USA).

## Results

### Patient population

During the study period, 6561 patients with severe sepsis including septic shock from 40 hospitals were documented. Of these, 1266 had no blood cultures drawn at the time of sepsis onset and 691 had no PCT measured, resulting in 4858 patients with blood culture results and a PCT value available for analysis (Table 1).

**Table 1:** Patient population regarding blood cultures and procalcitonin measurements

Procalcitonin measured	Blood cultures drawn before or after first dose of antibiotics			Total
	No	Yes, before	Yes, after	
No	254	281	156	691
Yes	1012	**3157**	**1701**	5870
Total	1266	3438	1857	6561

In sum, 4858 patients (marked in bold) with blood cultures and procalcitonin taken at sepsis onset were available for analysis

Baseline and outcome clinical information depending on blood culture results is presented in Table 2. PCT concentrations were about three times higher in patients with positive blood cultures compared to negative or contaminated blood cultures (*p* < 0.001). Those with positive blood cultures showed a slightly higher severity of disease (SOFA score, serum lactate, urine output) but a similar proportion of patients meeting the criteria for septic shock. There was no difference in PCT values between patients with negative or contaminated blood cultures (*p* = 0.8).

**Table 2:** Patient characteristics

Characteristic	All patients (*n* = 4858)	Blood culture results		
		Negative or contamination (*n* = 2875)	Positive with pathogen (*n* = 1983)	*p* value
Age (years)	70 (59–77)	70 (59–77)	70 (59–77)	0.37
Sex (male)	3060 (63)	1817 (63)	1243 (63)	0.71
Origin of infection				< 0.001
Community acquired	2136 (44.0)	1172 (39.6)	964 (46.5)	
Nosocomial (ICU)	1005 (20.7)	644 (24.0)	361 (19.2)	
Nosocomial (ward)	1579 (32.5)	990 (34.6)	589 (31.1)	
Nosocomial (nursing home)	138 (2.8)	69 (1.8)	69 (3.2)	
Focus of infection				
Respiratory	2035 (41.9)	1386 (48.2)	649 (32.7)	< 0.001
Abdominal	1632 (33.6)	1040 (36.2)	592 (29.9)	< 0.001
Urogenital	744 (15.3)	288 (10.0)	456 (23.0)	< 0.001
Bones/soft tissue	565 (11.6)	282 (9.8)	283 (14.3)	< 0.001
Other	673 (13.9)	300 (10.4)	373 (18.8)	< 0.001
Clinical data and scores				
PCT (ng/ml)	6.4 (1.7–25.6)	4.1 (1.2–15.5)	12.6 (3.3–41.9)	< 0.001
CRP (mg/ml)	208 (116–301)	196 (111–293)	219 (125–310)	< 0.001
WBC (10^9^)	16.3 (10.7–23.5)	16.1 (10.9–23.1)	16.4 (10.2–23.8)	0.85
Leukopenia (WBC ≤ 4)	545 (11.2)	281 (9.8)	264 (13.3)	< 0.001
Temperature (°C)	38.0 (36.3–38.8)	37.9 (36.0–38.7)	38.2 (36.8–39.0)	< 0.001
Lactate (mmol/l)	2.7 (1.6–5.0)	2.6 (1.5–4.7)	3.0 (1.7–5.5)	< 0.001
Urine output (ml/24 h)	1390 (620–2350)	1420 (660–2430)	1320 (580–2300)	0.02
SOFA	9 (7–12)	9 (7–11)	10 (7–12)	< 0.001
Septic shock^a^	2714 (55.9)	1577 (54.9)	1137 (57.3)	0.09
Mortality				
ICU mortality	1485 (30.6)	872 (30.4)	613 (30.9)	0.68
28-Day mortality	1562 (33.0)	888 (31.7)	674 (34.8)	0.03
Hospital mortality	1861 (38.4)	1080 (37.6)	781 (39.4)	0.21

Data expressed as median (Q1–Q3) or number (percentage). *p* values for comparison between negative and positive blood culture result groups

*ICU* intensive care unit, *PCT* procalcitonin, *CRP* C-reactive protein, *WBC* white blood cell count, *SOFA* Sequential Organ Failure Assessment Score

^a^Septic shock by Sepsis-3 criteria

### Prediction of Gram-negative bacteremia

Blood cultures positive with Gram-negative pathogens were found in 815 patients only, while 948 patients were blood culture-positive with Gram-positive bacteria, solely, and 65 with candida spp. only. Those 159 patients with any mix of the three groups of pathogens were excluded from the analysis.

**Fig. 1:**
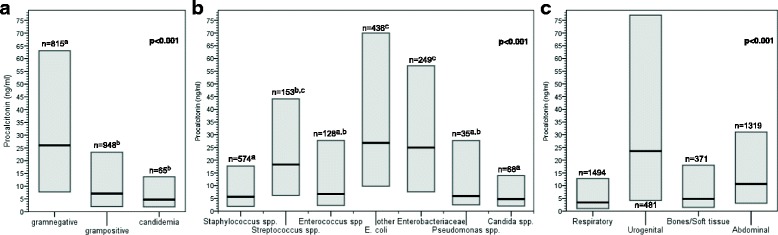
Initial PCT concentrations (ng/ml, median and IQR) associated with Gram stain (**a**) or type of pathogen (**b**) detected in blood culture drawn at sepsis onset, or associated with focus of infection (**c**). Significantly different (*p* < 0.001) in all three comparisons. n denotes number of cases represented by each bar and superscript letters denote homogeneous subsets

PCT concentrations in patients with Gram-negative bacteremia (26 ng/ml (7.7–63.1)) were distinctly higher than in patients with Gram-positive bacteremia (7.1 ng/ml (2.0–23.3)) or candidemia (4.7 ng/ml (1.9–13.7)) (*p* < 0.001) (Fig. 1a). There was no difference in C-reactive protein (CRP) concentrations (*p* = 0.7) or white blood cell count (*p* = 0.3) between the three groups.

The AUC in the ROC analysis was 0.69 (95% confidence interval 0.67–0.72) for the differentiation of Gram-negative bacteremia from Gram-positive bacteremia or candidemia and was 0.72 (95% CI 0.71–0.74) for the prediction of Gram-negative bacteremia compared to all other blood culture results including negative blood cultures (see Additional file 2: Figure S2). Test performances for optimal cutoff values are presented in Table 3.

**Table 3:** Test performance of procalcitonin for prediction of Gram-negative bacteremia

Classification	Optimal cutoff (ng/ml)	SENS	SPEC	PPV	NPV	PLR	NLR	ACC
Gram-negative bacteremia vs Gram-positive/candidemia	17.5	0.59	0.70	0.61	0.68	1.98	0.59	0.65
Gram-negative bacteremia vs all other blood culture results	10	0.69	0.65	0.33	0.9	1.97	0.47	0.66

Optimal cutoff values derived from receiver operating characteristics by Youden’s index and sensitivity (SENS), specificity (SPEC), positive predictive value (PPV), negative predictive value (NPV), positive likelihood ratio (PLR), negative likelihood ratio (NLR) and accuracy (ACC) calculated from the resulting 2 × 2 tables

### Pathogen species, foci of infection and PCT concentrations

Blood cultures positive with pathogens of a single group out of the predefined seven groups according to phylogenetic relationship and case numbers were found in 1643 patients (Table 3). We excluded 143 cases with pathogens which could not reasonably be grouped due to rarity and no close phylogenetic relationship to other pathogens. Another 207 patients with several groups of pathogens detected were excluded from the analysis. PCT concentrations differed significantly between the seven pathogen species (*p* < 0.001), with highest concentrations in *E. coli*, *Streptococcus* spp. and other *Enterobacteriaceae* (Fig. 1b). There were no differences in PCT concentrations within the groups of pathogens except for Staphylococci and no distinct subsets could be identified from the different types of Staphylococci (Table 4).

**Table 4:** Procalcitonin values associated with different pathogens

Pathogen species detected from blood cultures	Number	PCT (IQR)	*p* value
***Staphylococcus*** **spp.**	574	5.6 (1.9–17.7)	0.015
*Staphylococcus aureus*, methicillin sensitive	272	7.2 (2.7–20.7)	
*Staphylococcus aureus*, methicillin resistant	58	4.7 (1.4–10.6)	
Coagulase-negative Staphylococci, methicillin sensitive	151	5.2 (1.4–16.1)	
Coagulase-negative Staphylococci, methicillin resistant	65	3.8 (1.3–12.8)	
***Streptococcus*** **spp.**	153	18.2 (6.2–44.1)	0.12
Streptococci, Group A, B, C or G	59	18.2 (8.4–47.3)	
*Streptococcus pneumoniae*	63	21.6 (7–48.3)	
Other Streptococci (*Streptococcus mutans*, other viridans Streptococci)	26	6.8 (3.6–44.1)	
***Enterococcus*** **spp.**	128	6.8 (2.2–27.8)	0.5
*Enterococcus faecalis*	41	8.7 (2.1–54)	
*Enterococcus faecium*	71	6.7 (2.3–25.3)	
Vancomycin-resistant Enterococci	7	1.9 (0.5–99.3)	
***Escherichia coli***	436	26.8 (9.8–70)	
***Enterobacteriaceae*** **other than** ***E. coli***	249	24.9 (7.6–57.1)	0.07
*Enterobacter* spp*.*	38	21.1 (7.6–56.8)	
*Klebsiella* spp*.*	123	21.5 (6–49.7)	
*Proteus* spp*.*	47	46.8 (9.1–97.6)	
*Serratia* spp*.*	20	12.1 (6.5–42.4)	
*Citrobacter* spp*.*	9	37.8 (15.1–113.1)	
*Enterobacteriaceae*, other	2	3.7 (2.4–5)	
***Pseudomonas*** **spp.**	35	5.9 (2.1–28.4)	0.4
*Pseudomonas aeruginosa*	32	6.1 (3.1–30.6)	
*Pseudomonas*, other	3	2.0 (0.6–28.4)	
***Candida spp.***	68	4.7 (2–14)	0.7
*Candida albicans*	57	5.3 (2.1–15.3)	
*Candida*, other	37	6.5 (2.2–17.4)	
**Rare pathogens**			
*Listeria monocytogenes*	7	8.0 (0.8–19.7)	
*Acinetobacter* spp*.*	7	5.8 (0.9–39.0)	
*Haemophilus* spp*.*	4	29.9 (17.3–36.6)	

Absolute numbers for defined groups of pathogens (in bold) and the pathogens composing them (reported only for cases with a single pathogen detected from blood culture). *p* values for differences between pathogens within a group. Additionally, three most frequent of the rare pathogens reported at end of the table

*IQR* interquartile range, *PCT* procalcitonin

Within the four infection categories, 3665 patients had a single focus of infection (Fig. 1c). We excluded 469 cases with unknown or rare focus of infection and 723 cases with more than one focus. There were significant differences in PCT concentrations between the different foci of infection (*p* < 0.001). PCT concentrations were highest in patients with urogenital infection, followed by abdominal infection, and lowest in respiratory infection (Fig. 1c).

A linear regression model was calculated based on 1146 cases with blood cultures positive with pathogens of a single group of pathogens and a single focus of infection using pathogen group and focus of infection as predictors of logPCT (Table 5). Streptococci, *E. coli* and other *Enterobacteriaceae* were associated with three times higher PCT values than other pathogens in blood culture. Urogenital or abdominal foci of infection were associated with a twofold increased PCT concentration independent from the detected pathogen. There was no significant interaction effect between detected pathogen and focus of infection for the association with PCT concentrations.

**Table 5:** Linear regression model limited to cases with one pathogen group and one focus of infection

Variable	Regression coefficient	95% CI	*p* value	Multiplier (95% CI)
*Staphylococcus* spp.	0.00	(− 0.22 to 0.21)	1.0	1.0 (0.6–1.6)
*Streptococcus* spp.	0.50	(0.26–0.74)	< 0.001	3.2 (1.8–5.5)
*Enterococcus* spp.	0.06	(−0.18 to 0.31)	0.6	1.1 (0.7–2.0)
*Escherichia coli*	0.50	(0.29–0.72)	< 0.001	3.2 (1.9–5.2)
*Enterobacteriaceae*, other	0.49	(0.26–0.72)	< 0.001	3.1 (1.8–5.2)
*Pseudomonas* spp.	0.04	(− 0.3 to 0.37)	0.8	1.1 (0.5–2.3)
*Candida* spp.	Reference			Reference
Respiratory	− 0.01	(− 0.14 to 0.12)	0.9	1 (0.7–1.3)
Abdominal	0.27	(0.14–0.41)	< 0.001	1.9 (1.4–2.6)
Urogenital	0.32	(0.17–0.47)	< 0.001	2.1 (1.5–3.0)
Bones/soft tissue	Reference			Reference
Intercept	0.63	(0.41–0.86)	< 0.001	4.3 (2.6–7.2)

Stepwise linear regression model for influence of pathogens in blood culture and focus of infection on logPCT (*p* < 0.001 for both steps) limited to 1146 cases with one pathogen group detected in blood culture and one focus of infection; adjusted *R*^2^ = 0.18; effect of the interaction term not significant (*p* = 0.13) and it was omitted from final model

After reversal of the logarithmic transformation, the multiplier equals 10^regression coefficient^, resulting in PCT_predicted_ = 4.3 × pathogen × focus × error

*CI* confidence interval, *PCT* procalcitonin

A second linear regression model was calculated based on all cases showing that the detection of any pathogen except *Pseudomonas* spp. and *Candida* spp. in blood cultures was associated with higher PCT concentrations compared to negative blood cultures as the reference group (Table 6).

**Table 6:** Linear regression model for all cases

Variable	Regressioncoefficient	95% CI	*p* value	Multiplier (95% CI)
*Staphylococcus* spp.	0.13	(0.06–0.2)	< 0.001	1.4 (1.2–1.6)
*Streptococcus* spp.	0.59	(0.47–0.71)	< 0.001	3.9 (3.0–5.1)
*Enterococcus* spp	0.17	(0.03–0.29)	0.01	1.5 (1.1–2.0)
*Escherichia coli*	0.62	(0.54–0.7)	< 0.001	4.2 (3.5–5.)
*Enterobacteriaceae*, other	0.56	(0.46–0.65)	< 0.001	3.7 (2.9–4.6)
*Pseudomonas* spp.	0.23	(− 0.02 to 0.47)	0.07	1.7 (1.0–2.9)
*Candida* spp.	0.07	(− 0.11 to 0.24)	0.44	1.2 (0.8–1.8)
Several pathogens	0.46	(0.36–0.56)	< 0.001	2.9 (2.3–3.7)
Rare pathogens	0.28	(0.16–0.4)	< 0.001	1.9 (1.5–2.6)
No pathogen detected	Reference			Reference
Respiratory	−0.24	(− 0.32 to – 0.16)	< 0.001	0.6 (0.5–0.7)
Abdominal	0.14	(0.06–0.22)	< 0.001	1.5 (1.3–1.8)
Urogenital	0.18	(0.08–0.27)	< 0.001	1.6 (1.3–2.0)
Bones/soft tissue	− 0.17	(− 0.27 to – 0.07)	< 0.001	0.8 (0.6–1.0)
Several foci	−0.13	(− 0.22 to – 0.04)	< 0.001	0.8 (0.7–1.0)
Other/unknown	Reference			Reference
Intercept	0.70	(0.62–0.77)	< 0.001	4.5 (3.9–5.3)

Stepwise linear regression for influence of pathogens in blood culture and focus of infection on logPCT (*p* < 0.001 for both steps) including all 4857 cases with PCT measurement and blood cultures taken; adjusted *R*^2^ = 0.15; effect of the interaction term not significant (*p* = 0.47) and it was omitted from the final model

After reversal of the logarithmic transformation, the multiplier equals 10^regression coefficient^, resulting in PCT_predicted_ = 4.5 × pathogen × focus × error

*CI* confidence interval, *PCT* procalcitonin

**Fig. 2:**
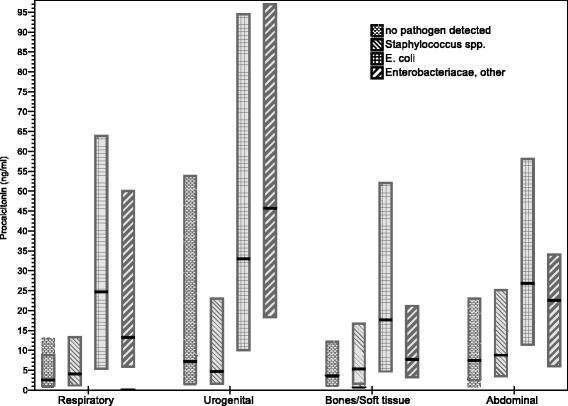
Median and IQR of PCT concentrations associated with focus of infection in combination with most frequent blood culture results. Associations of focus and pathogens independent from each other (see Tables 5 and 6)

The effects of focus of infection on PCT serum concentrations stratified for the most frequent pathogens are shown in Fig. 2. Including potential confounders such as age, sex, origin of infection, type of ICU admission and disease severity (SOFA score, septic shock and lactate) as additional predictors (Table 7) showed very similar effects for pathogens and foci of infection. Also, restricting the analyses to cases that had blood cultures drawn before the start of a new antimicrobial therapy (see Additional file 2: Tables S1 and S2) did not result in major changes.

**Table 7:** Linear regression model for all cases with forward selection of potential confounders

Variable	Regression coefficient	95% CI	*p* value	Multiplier (95% CI)
*Staphylococcus* spp.	0.11	(0.05–0.18)	<.001	1.3 (1.1–1.5)
*Streptococcus* spp.	0.50	(0.39–0.62)	<.001	3.2 (2.5–4.2)
*Enterococcus* spp.	0.14	(0.02–0.26)	0.02	1.4 (1.0–1.8)
*Escherichia coli*	0.52	(0.45–0.59)	<.001	3.3 (2.8–3.9)
Enterobacteriacae, other	0.51	(0.42–0.6)	<.001	3.2 (2.6–4.0)
*Pseudomonas* spp.	0.16	(−0.06 to 0.39)	0.16	1.4 (0.9–2.5)
*Candida* spp.	0.14	(− 0.03 to 0.31)	0.11	1.4 (0.9–2.0)
Several pathogens	0.36	(0.26–0.46)	<.001	2.3 (1.8–2.9)
Rare pathogens	0.20	(0.08–0.32)	<.001	1.6 (1.2–2.1)
No pathogen detected	Reference			Reference
Respiratory	−0.24	(−0.31 to – 0.16)	<.001	0.6 (0.5–0.7)
Abdominal	0.09	(0.01–0.17)	<.001	1.2 (1.0–1.5)
Urogenital	0.20	(0.11–0.3)	0.02	1.6 (1.3–2.0)
Bones/soft tissue	−0.19	(− 0.28 to – 0.1)	<.001	0.6 (0.5–0.8)
Several foci	− 0.10	(− 0.19 to – 0.02)	<.001	0.8 (0.6–1.0)
Other/unknown	Reference			Reference
Male	0.06	(0.02–0.1)	0.002	1.1 (1.0–1.3)
Female	Reference			Reference
Community acquired	0.16	(0.11–0.2)	<.001	1.4 (1.3–1.6)
ICU acquired	−0.14	(−0.2 to – 0.09)	<.001	0.7 (0.6–0.8)
Normal ward acquired	Reference			Reference
Lactate per mmol/l	0.01	(0.01–0.02)	<.001	1.0 (1.0–1.0)
SOFA score per point	0.03	(0.02–0.04)	<.001	1.1 (1.0–1.1)
Septic shock	0.22	(0.18–0.27)	<.001	1.7 (1.5–1.9)
Intercept	0.66	(0.57–0.74)	<.001	4.5 (3.7–5.5)

General linear model for influence of pathogens in blood culture and focus of infection on logPCT (*p* < 0.001 for both factors) including all 3156 cases with PCT measurement and blood cultures taken before start of new antimicrobial therapy; additional potential confounders added by stepwise selection; age (*p* = 0.3) and type of ICU admission (0.14) were only ones not selected. Adjusted *R*^2^ = 0.24; interaction terms not included in model

After reversal of the logarithmic transformation, the multiplier equals 10^regression coefficient^, resulting in PCT_predicted_ = 4.5 × pathogen × focus × confounders × error

*CI* confidence interval, *ICU* intensive care unit, *PCT* procalcitonin, *SOFA* Sequential Organ Failure Assessment Score

## Discussion

The main finding of this study is confirmation that sepsis patients with proven Gram-negative bacteremia have significantly higher PCT concentrations than patients with Gram-positive bacteremia or candidemia. However, the present study demonstrates that the diagnostic accuracy is not sufficient to tailor empiric antimicrobial therapy based on PCT concentrations. In addition, for the first time in a large number of clinically well-characterized patients, an independent association of focus of infection and specific groups of pathogens with differences in PCT concentrations was detected. Urogenital or abdominal foci of infection were associated with two times higher PCT values, and Streptococci, *E. coli* and other *Enterobacteriaceae* detected from BC with three times higher PCT values.

The association of Gram-negative bacteremia with high PCT concentrations is in agreement with other studies performed in different patient populations [5, 8–10, 13–15]. However, absolute PCT concentrations reported in Gram-negative bacteremia differed considerably between these studies with median values ranging from 2.2 ng/ml [10] up to 39 ng/ml [8]. These differences are most likely attributable to different patient populations including patients with endocarditis [13] and febrile neutropenia [14]. Some larger patient cohorts were composed of all patients with blood culture results and corresponding PCT measurements available from intensive care units [5] or whole hospitals [9, 10] lacking clinical data including any confirmed diagnosis of infection or sepsis. Hence, comparability to our results is limited. Studies reporting patients diagnosed with clinical sepsis diagnosis and bacteremia, resembling our patient population, reported median PCT concentrations of 39 ng/ml [8] and 27 ng/ml [16] in patients with Gram-negative bacteremia, being similar to the median PCT concentration of 26 ng/ml in 815 patients with Gram-negative bacteremia in our study.

As PCT expression is at least partly induced by inflammatory cytokines [17], differences in the pathogen specific signaling could explain the observed differences in PCT concentrations in the present study. Lipopolysaccharides (LPS), cell wall components of Gram-negative bacteria, are the prototypical class of pathogen-associated molecular patterns (PAMPs) and are recognized by cells of the innate immune system via toll-like receptor 4 (TLR4), while lipoteichoic acid (LTA), a cell wall component of Gram-positive bacteria, is recognized by toll-like receptor 2 (TLR2) [18, 19]. The intracellular signaling cascades of both receptors converge in part through common adaptor molecules on the same transcription factors, and both receptors are activated by components of whole Gram-negative and Gram-positive bacteria [18]. Nevertheless, the TLR4-dominant activation by Gram-negative bacteria and the TLR2-dominant activation by Gram-positive bacteria result in a very different induction of several inflammatory cytokines [18, 20–22] and different gene expression patterns in leukocytes [20] in vitro. Higher levels of Interleukin-6 (IL-6) [23, 24] and Interleukin-8 (IL-8) [24] have been reported in patients with Gram-negative bacteremia. These differences probably contribute to the observed differences in PCT response seen with Gram-negative or Gram-positive bacteremia.

To our knowledge, we are the first to describe significant differences in PCT concentrations for more specific groups of pathogens in a high number of clinically well-defined patients. Previous reports have been difficult to interpret due to the small number of cases for any but the most common pathogens [5, 16]. In two large studies, one group reported nearly identical PCT concentrations for Staphylococci, Streptococci and Enterococci [10], and the other reported higher PCT concentrations in Streptococci than in other Gram-positive bacteria, but those PCT concentrations were still much lower than in the reported groups of *Enterobacteriaceae* [9]. Both studies had heterogeneous patient populations with extremely limited clinical information and did not assess the clinical relevance of the detected pathogens. A recent study on community-acquired pneumonia showed higher PCT concentrations associated with typical bacteria than with atypical bacteria [25]. One experimental study described several differences in intracellular signaling after TLR activation between Group A Streptococci, *Staphylococcus aureus* and *E. coli*, and speculated that this results in a lower inflammatory response in Streptococci [26]. Another in-vitro study compared cytokine production induced in cord blood cells by heat-killed Group B Streptococci, *E. coli* and *Staphylococcus epidermidis*, and found quite different response patterns with higher cytokine levels for *E. coli* and Streptococci [21]. The cytolytic toxin pneumolysin of *Streptococcus pneumoniae* is known to strongly activate TLR 4 [27], which may contribute to the high concentrations of PCT seen with Streptococcal bacteremia in our study.

The difference in the clinical course of sepsis depending on the focus of infection is widely acknowledged [28]. Differences in mortality depending on the focus of infection have been reported previously [29]. To our knowledge, we are the first to systematically assess the association of different foci of infection in sepsis with measured PCT concentrations in ICU patients. One recent study investigating emergency room patients with clinical diagnosis of severe sepsis and septic shock and low PCT concentrations (< 0.25 ng/ml) found a significant association of pneumonia with low PCT compared to abdominal sepsis, even after correcting for multiple factors including bacteremia [30]. Such a difference in the PCT response might be explained by differences in the spectrum of pathogens depending on the focus of infection. However, the focus of infection and the underlying pathogen were independently associated with PCT concentrations in our multivariate analysis. Therefore, variations in the host response or in rates of bacteremia depending on the site of infection could be another explanation for differences in PCT concentrations. Indeed, bacteremia with a low bacterial load developed late in the course of experimental pneumonia [31]. Higher degrees of systemic bacterial load in bacteremia patients seemed to be associated with higher PCT concentrations [32]. An abdominal focus might be associated with a higher bacterial load as the lymphatic flow from the peritoneal cavity drains directly to the systemic circulation and the venous blood from the abdominal organs drains to the liver. To our knowledge there are no experimental or clinical data helping to support or discard those hypotheses. In mice, a gene array study comparing a pneumococcal pneumonia model and a polymicrobial fecal peritonitis model could not differentiate effects depending on sites of infection and pathogens [33].

Several authors have suggested that very high PCT concentrations are associated with Gram-negative bacteremia, and thus to tailor antimicrobial therapy [5–10, 15, 16]. However, according to our data, the clinical use of such an approach is limited by several factors. Firstly, a sepsis patient with only moderately elevated PCT concentrations has a low probability of Gram-negative bacteremia, but might still have a severe Gram-negative infection without bacteremia. Secondly, there is a large heterogeneity in PCT concentrations leaving a large area of overlap between Gram-negative bacteremia, Gram-positive bacteremia and candidemia. Although the reported AUCs for the differentiation of Gram-negative bacteremia from Gram-positive bacteremia were between 0.77 and 0.87 [5, 8, 9], we could not confirm such a high diagnostic accuracy as our analysis resulted in an AUC of 0.69 only. Furthermore, specificity was insufficient for clinical application. A diagnostic test guiding decisions about potentially lifesaving therapy needs to have a better diagnostic accuracy than that observed in our data in a high number of patients. Thirdly, as demonstrated in our study, the observed differences in PCT concentrations are associated with more specific groups of pathogens. We observed no differences in PCT concentrations between bacteremia with *Staphylococcus* spp., *Enterococcus* spp., *Pseudomonas* spp. and *Candida* spp., four groups of pathogens that would require very different empirical antimicrobial therapy.

Our analysis has several strengths and weaknesses. To our knowledge, this is the first study on the subject combining a large number of cases with a sufficient amount of clinical data in a fairly homogeneous group of sepsis patients treated in intensive care units. In contrast to previous studies, mostly based on laboratory data only, all of our patients had a clinical diagnosis of sepsis with organ dysfunction and the clinical relevance of any detected pathogen was assessed by the treating physicians. As this is a secondary analysis of data from a multicenter quality improvement trial, the available information is limited. We have no information on how the focus of infection was diagnosed and which pathogens were detected from the focus of infection. Assessment of the blood culture results and the underlying infection was not confirmed by an independent review board.

## Conclusions

Serum procalcitonin concentrations are higher in patients with Gram-negative bacteremia than in patients with Gram-positive bacteremia or candidemia. However, the discriminatory power of this difference is too low to guide therapeutic decisions. Variations in PCT serum concentrations are not determined by Gram-negative or Gram-positive bacteria per se, but by certain groups of pathogens and different foci of infection. Although PCT concentrations could give some additional information about the likelihood of distinct pathogens and foci of infection in sepsis patients, this information should be interpreted with caution and in due consideration with all available clinical information, and it can never replace a thorough microbiological work up and search for a focus of infection. However, it might reflect the degree of immune response to the underlying infection.

## Additional files

Additional file 1: All involved ethical bodies with reference number of the vote (PDF 45 kb)

Additional file 2: **Figure S1.** P–P plots of PCT and logPCT. **Figure S2.** AUC plots for ROC analyses. **Tables S1** and **S2.** Regression models limited to cases with blood cultures taken before start of antimicrobial therapy (DOC 215 kb)

## Abbreviations

ANOVA: Analysis of variance
AUC: Area under the curve
CI: Confidence interval
CRP: C-reactive protein
ICU: Intensive care unit
IQR: Interquartile range
PCT: Procalcitonin
ROC: Receiver operating characteristic
SOFA: Sequential Organ Failure Assessment Score
spp.: Species pluralis
WBC: White blood cell count

## References

[1] Maruna P, Nedelnikova K, Gurlich R (2000). Physiology and genetics of procalcitonin. Physiol Res.

[2] Meisner M (2002). Pathobiochemistry and clinical use of procalcitonin. Clin Chim Acta.

[3] Wacker C, Prkno A, Brunkhorst FM, Schlattmann P (2013). Procalcitonin as a diagnostic marker for sepsis: a systematic review and meta-analysis. Lancet Infect Dis.

[4] Rhodes A, Evans LE, Alhazzani W, Levy MM, Antonelli M, Ferrer R, Kumar A, Sevransky JE, Sprung CL, Nunnally ME (2017). Surviving Sepsis Campaign: International Guidelines for Management of Sepsis and Septic Shock: 2016. Intensive Care Med.

[5] Brodska H, Malickova K, Adamkova V, Benakova H, Stastna MM, Zima T (2012). Significantly higher procalcitonin levels could differentiate Gram-negative sepsis from Gram-positive and fungal sepsis. Clin Exp Med.

[6] Moyer MW (2012). New biomarkers sought for improving sepsis management and care. Nat Med.

[7] Beloborodova NV, Vostrikova T, Chernevskaia EA (2008). Etiology of postoperative bacteremias in an intensive care unit: an association with the level of procalcitonin. Anesteziol Reanimatol.

[8] Charles PE, Ladoire S, Aho S, Quenot JP, Doise JM, Prin S, Olsson NO, Blettery B (2008). Serum procalcitonin elevation in critically ill patients at the onset of bacteremia caused by either Gram negative or Gram positive bacteria. BMC Infect Dis.

[9] Leli C, Ferranti M, Moretti A, Al Dhahab ZS, Cenci E, Mencacci A (2015). Procalcitonin levels in gram-positive, gram-negative, and fungal bloodstream infections. Dis Markers.

[10] Oussalah A, Ferrand J, Filhine-Tresarrieu P, Aissa N, Aimone-Gastin I, Namour F, Garcia M, Lozniewski A, Gueant JL (2015). Diagnostic accuracy of procalcitonin for predicting blood culture results in patients with suspected bloodstream infection: an observational study of 35,343 consecutive patients (a STROBE-compliant article). Medicine (Baltimore).

[11] Bloos F, Ruddel H, Thomas-Ruddel D, Schwarzkopf D, Pausch C, Harbarth S, Schreiber T, Grundling M, Marshall J, Simon P (2017). Effect of a multifaceted educational intervention for anti-infectious measures on sepsis mortality: a cluster randomized trial. Intensive Care Med.

[12] Bloos F, Thomas-Ruddel D, Ruddel H, Engel C, Schwarzkopf D, Marshall JC, Harbarth S, Simon P, Riessen R, Keh D (2014). Impact of compliance with infection management guidelines on outcome in patients with severe sepsis: a prospective observational multi-center study. Crit Care.

[13] Kocazeybek B, Kucukoglu S, Oner YA (2003). Procalcitonin and C-reactive protein in infective endocarditis: correlation with etiology and prognosis. Chemotherapy.

[14] Koivula I, Hamalainen S, Jantunen E, Pulkki K, Kuittinen T, Nousiainen T, Juutilainen A (2011). Elevated procalcitonin predicts Gram-negative sepsis in haematological patients with febrile neutropenia. Scand J Infect Dis.

[15] Nakajima A, Yazawa J, Sugiki D, Mizuguchi M, Sagara H, Fujisiro M, Shibazaki M, Hitani A, To M, Haruki K (2014). Clinical utility of procalcitonin as a marker of sepsis: a potential predictor of causative pathogens. Intern Med.

[16] Guo SY, Zhou Y, Hu QF, Yao J, Wang H (2015). Procalcitonin is a marker of Gram-negative bacteremia in patients with sepsis. Am J Med Sci.

[17] Matwiyoff GN, Prahl JD, Miller RJ, Carmichael JJ, Amundson DE, Seda G, Daheshia M (2012). Immune regulation of procalcitonin: a biomarker and mediator of infection. Inflamm Res.

[18] Gao H, Evans TW, Finney SJ (2008). Bench-to-bedside review: sepsis, severe sepsis and septic shock—does the nature of the infecting organism matter?. Crit Care.

[19] Leaver S, Burke Gaffney A, Evans TW, Vincent J-L (2008). Gram-positive and Gram-negative sepsis: two disease entities?. Intensive Care Medicine: Annual Update 2008.

[20] Feezor RJ, Oberholzer C, Baker HV, Novick D, Rubinstein M, Moldawer LL, Pribble J, Souza S, Dinarello CA, Ertel W (2003). Molecular characterization of the acute inflammatory response to infections with gram-negative versus gram-positive bacteria. Infect Immun.

[21] Mohamed MA, Cunningham-Rundles S, Dean CR, Hammad TA, Nesin M (2007). Levels of pro-inflammatory cytokines produced from cord blood in-vitro are pathogen dependent and increased in comparison to adult controls. Cytokine.

[22] Re F, Strominger JL (2001). Toll-like receptor 2 (TLR2) and TLR4 differentially activate human dendritic cells. J Biol Chem.

[23] Abe R, Oda S, Sadahiro T, Nakamura M, Hirayama Y, Tateishi Y, Shinozaki K, Hirasawa H (2010). Gram-negative bacteremia induces greater magnitude of inflammatory response than Gram-positive bacteremia. Crit Care.

[24] Prat C, Dominguez J, Andreo F, Blanco S, Pallares A, Cuchillo F, Ramil C, Ruiz-Manzano J, Ausina V (2006). Procalcitonin and neopterin correlation with aetiology and severity of pneumonia. J Inf Secur.

[25] Self WH, Balk RA, Grijalva CG, Williams DJ, Zhu Y, Anderson EJ, Waterer GW, Courtney DM, Bramley AM, Trabue C (2017). Procalcitonin as a marker of etiology in adults hospitalized with community-acquired pneumonia. Clin Infect Dis.

[26] Wu S, Ma C, Gao X, Zhang L, Miao Q, Li M, Li W, Song X, Wang X, Liu J (2016). Group A Streptococcus induces less p65 nuclear translocation and non-classical nuclear factor kappa B activation in macrophages, which possibly leads to a weaker inflammatory response. Int J Infect Dis.

[27] Malley R, Henneke P, Morse SC, Cieslewicz MJ, Lipsitch M, Thompson CM, Kurt-Jones E, Paton JC, Wessels MR, Golenbock DT (2003). Recognition of pneumolysin by Toll-like receptor 4 confers resistance to pneumococcal infection. Proc Natl Acad Sci U S A.

[28] Vincent JL, Opal S, Torres A, Bonten M, Cohen J, Wunderink R (2003). The PIRO concept: I is for infection. Crit Care.

[29] Osborn TM, Phillips G, Lemeshow S, Townsend S, Schorr CA, Levy MM, Dellinger RP (2014). Sepsis severity score: an internationally derived scoring system from the surviving sepsis campaign database. Crit Care Med.

[30] Choe EA, Shin TG, Jo IJ, Hwang SY, Lee TR, Cha WC, Sim MS (2016). The prevalence and clinical significance of low procalcitonin levels among patients with severe sepsis or septic shock in the emergency department. Shock.

[31] Andonegui G, Goring K, Liu D, McCafferty DM, Winston BW (2009). Characterization of S. pneumoniae pneumonia-induced multiple organ dysfunction syndrome: an experimental mouse model of gram-positive sepsis. Shock.

[32] van Nieuwkoop C, Bonten TN, van't Wout JW, Kuijper EJ, Groeneveld GH, Becker MJ, Koster T, Wattel-Louis GH, Delfos NM, Ablij HC (2010). Procalcitonin reflects bacteremia and bacterial load in urosepsis syndrome: a prospective observational study. Crit Care.

[33] Weber M (2010). Transkriptomik der Inflammation an der Maus: die Lunge als Ausgangsorgan und als Zielorgan der Sepsis.

[34] Thomas-Rueddel DO, Poidinger B, Eiche J, Jelschen F, Kott M, Weiss M, Reinhart K, Bloos F (2015). Influence of pathogen and focus of infection on procalcitonin values in bacteremic severe sepsis. Infection.

