# Supplementary material for: Influence of pathogen and focus of infection on procalcitonin values in sepsis patients with bacteremia or candidemia
Source: Crit Care. 2018 May 13;22:128. doi: 10.1186/s13054-018-2050-9 (PMC5949148; doi:10.1186/s13054-018-2050-9)
Supplement: Supplementary file 1 — All involved ethical bodies with reference number of the vote (PDF 45 kb) [file 13054_2018_2050_MOESM1_ESM.pdf]

Additional File 1– Involved Ethical Bodies

- Ethics committee of the University Hospital Jena (2910–08/10)
- Ethics committee of the “Ärztekammer Nordrhein” (2010403)
- Ethics committee of the “Ärztekammer Westfalen-Lippe” (2010-518-b-S)
- Ethics committee of the “Landesärztekammer Baden-Würtemberg” (B-F-2010-056)
- Ethics committee of the “Landesärztekammer Bayern” (7/10284)
- Ethics committee of the “Landesärztekammer Hessen” (MC 245/2010)
- Ethics committee of the “Landesärztekammer Niedersachsen” (Ar/211/2010)
- Ethics committee of the “Landesärztekammer Saarland” (195/10)
- Ethics committee of the “Landesärztekammer Sachsen” (EK-BR—51/10-1)
- Ethics committee of the Medical Faculty Leipzig (324-10-08112010)
- Ethics committee of the “Landesärztekammer Sachsen-Anhalt” (33/10)
- Ethics committee of the “Landesärztekammer Thüringen” (38831/2010/109)
- Ethics committee of the Medical Faculty Greifswald (BB 129/10)
- Ethics committee of the Medical Faculty Kiel (B 204/11)
- Ethics committee of the Medical Faculty Tübingen (556/2010BO2)
- Ethics committee of the University Ulm (295/10)
- Ethics committee of the University Witten-Herdecke (90/2010)
