# Supplementary material for: Influence of pathogen and focus of infection on procalcitonin values in sepsis patients with bacteremia or candidemia
Source: Crit Care. 2018 May 13;22:128. doi: 10.1186/s13054-018-2050-9 (PMC5949148; doi:10.1186/s13054-018-2050-9)
Supplement: Supplementary file 2 — Figure S1. P–P plots of PCT and logPCT. Figure S2. AUC plots for ROC analyses. Tables S1 and S2. Regression models limited to cases with blood cultures taken before start of antimicrobial therapy (DOC 215 kb) [file 13054_2018_2050_MOESM2_ESM.doc]

**Additional file 2 – Additional Tables and Figures**


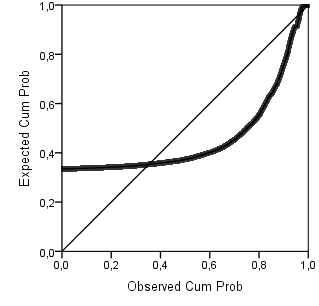

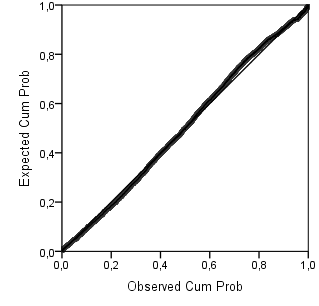


**PCT**

**logPCT**

**Figure S1:** Normal distribution P-P plots of Procalcitonin (PCT) and logarithmical transformed Procalcitonin (logPCT)

gram-negative vs. gram-positive & candidemia

gram-negative vs. all other

blood culture results

AUC=0.69 (0.67-0.72 95%-CI)

p<0.001

AUC=0.72 (0.71-0.74 95%-CI)

p<0.001

**Figure S2:** ROC analysis for the prediction of blood culture results by PCT

Table S1 Linear regression model - limited to cases with one pathogen group and one focus of infection with blood cultures before antimicrobial therapy

| **Variable** | **Regression Coefficient** | **95% Confidence Interval** | **p-Value** | **Multiplier (95% CI)** |
| --- | --- | --- | --- | --- |
| Staphylococcus spp. | -0.23 | (-0.56 - 0.1) | 0.18 | 0.6 (0.3 - 1.3) |
| Streptococcus spp. | 0.42 | (0.07 - 0.77) | 0.02 | 2.6 (1.2 - 5.9) |
| Enterococcus spp. | -0.13 | (-0.5 - 0.25) | 0.5 | 0.7 (0.3 - 1.8) |
| E. coli | 0.32 | (-0.01 - 0.65) | 0.06 | 2.1 (1 - 4.4) |
| Enterobacteriacae, other | 0.33 | (-0.01 - 0.67) | 0.06 | 2.1 (1 - 4.7) |
| Pseudomonas spp. | -0.16 | (-0.61 - 0.29) | 0.5 | 0.7 (0.2 - 1.9) |
| Candida spp. | reference |  |  | reference |
| respiratory | -0.13 | (-0.28 - 0.02) | 0.09 | 0.7 (0.5 - 1.1) |
| abdominal | 0.17 | (0.01 - 0.33) | 0.04 | 1.5 (1 - 2.2) |
| urogenital | 0.21 | (0.04 - 0.39) | 0.02 | 1.6 (1.1 - 2.4) |
| bones/Soft tissue | reference |  |  | reference |
| intercept | 0.91 | (0.56 - 1.25) | <0.001 | 8.1 (3.7 - 17.8) |

General linear model for the influence of pathogens in blood culture and focus of infection on logPCT (p<0.001 for both factors) limited to 842 cases with one pathogen group detected in blood culture and one focus of infection and with blood cultures taken before the start of a new antimicrobial therapy; adjusted R square 0.19; the effect of the interaction term was not significant (p=0.25) and it was omitted from the final model.
After reversal of the logarithmic transformation, the multiplier equals 10^regression coefficient^, resulting in PCT_predicted_=8.1x pathogen x focus x error’.

Table S2: Linear regression model - all cases with blood cultures before antimicrobial therapy

| **Variable** | **Regression Coefficient** | **95% Confidence Interval** | **p-Value** | **Multiplier (95% CI)** |
| --- | --- | --- | --- | --- |
| Staphylococcus spp. | 0.11 | (0.03 - 0.19) | 0.008 | 1.3 (1.1 - 1.5) |
| Streptococcus spp. | 0.67 | (0.54 - 0.8) | <.001 | 4.7 (3.4 - 6.4) |
| Enterococcus spp | 0.17 | (-0.01 - 0.35) | 0.06 | 1.5 (1 - 2.2) |
| E. coli | 0.62 | (0.53 - 0.71) | <.001 | 4.2 (3.4 - 5.1) |
| Enterobacteriacae, other | 0.57 | (0.46 - 0.69) | <.001 | 3.8 (2.9 - 4.9) |
| Pseudomonas spp. | 0.22 | (-0.07 - 0.51) | 0.14 | 1.6 (0.8 - 3.2) |
| Candida spp. | 0.14 | (-0.12 - 0.4) | 0.28 | 1.4 (0.8 - 2.5) |
| several pathogens | 0.49 | (0.37 - 0.6) | <.001 | 3.1 (2.3 - 4) |
| rare pathogens | 0.25 | (0.11 - 0.39) | 0.001 | 1.8 (1.3 - 2.4) |
| no pathogen detected | reference |  |  | reference |
| respiratory | -0.21 | (-0.3 - -0.12) | <.001 | 0.6 (0.5 - 0.8) |
| abdominal | 0.19 | (0.1 - 0.29) | <.001 | 1.6 (1.3 - 2) |
| urogenital | 0.19 | (0.07 - 0.3) | 0.001 | 1.5 (1.2 - 2) |
| bones/soft tissue | -0.1 | (-0.22 - 0.02) | 0.11 | 0.8 (0.6 - 1.1) |
| several foci | -0.12 | (-0.22 - -0.02) | 0.02 | 0.8 (0.6 - 1) |
| other/unknown | reference |  |  | reference |
| intercept | 0.66 | (0.57 - 0.74) | <.001 | 4.5 (3.7 - 5.5) |

General linear model for the influence of pathogens in blood culture and focus of infection on logPCT (p<0.001 for both factors) including all 3,156 cases with procalcitonin measurement and blood cultures taken before the start of a new antimicrobial therapy; adjusted R square 0.17; the effect of the interaction term was not significant (p=0.38) and it was omitted from the final model.
After reversal of the logarithmic transformation, the multiplier equals 10^regression coefficient^, resulting in PCT_predicted_=4.5 x pathogen x focus x error’.
